# Personality traits affect learning performance in dwarf goats (*Capra hircus*)

**DOI:** 10.3389/fvets.2022.916459

**Published:** 2022-07-15

**Authors:** Marie-Antonine Finkemeier, Annika Krause, Armin Tuchscherer, Birger Puppe, Jan Langbein

**Affiliations:** ^1^Institute of Behavioural Physiology, Research Institute for Farm Animal Biology (FBN), Dummerstorf, Germany; ^2^Behavioural Sciences, Faculty of Agricultural and Environmental Sciences, University of Rostock, Rostock, Germany; ^3^Institute of Genetics and Biometry, Research Institute for Farm Animal Biology (FBN), Dummerstorf, Germany

**Keywords:** discrimination learning, personality, cognition, open field test, novel object test, serial reversal, goats

## Abstract

A wide range of species exhibit time- and context-consistent interindividual variation in a number of specific behaviors related to an individual's personality. Several studies have shown that individual differences in personality-associated behavioral traits have an impact on cognitive abilities. The aim of this study was to investigate the relationship between personality traits and learning abilities in dwarf goats. The behavior of 95 goats during a repeated open field (OF) and novel object test (NO) was analyzed, and two main components were identified using principal component analysis: *boldness* and *activity*. In parallel, the goats learned a 4-choice visual initial discrimination task (ID) and three subsequent reversal learning (RL) tasks. The number of animals that reached the learning criterion and the number of trials needed (TTC) in each task were calculated. Our results show that goats with the lowest learning performance in ID needed more TTC in RL1 and reached the learning criterion less frequently in RL2 and RL3 compared to animals with better learning performance in ID. This suggests a close relationship between initial learning and flexibility in learning behavior. To study the link between personality and learning, we conducted two analyses, one using only data from the first OF- and NO-test (*momentary* personality traits), while the other included both tests integrating only animals that were stable for their specific trait (*stable* personality traits). No relationship between personality and learning was found using data from only the first OF- and NO-test. However, stability in the trait *boldness* was found to have an effect on learning. Unbold goats outperformed bold goats in RL1. This finding supports the general hypothesis that bold animals tend to develop routines and show less flexibility in the context of learning than unbold individuals. Understanding how individual personality traits can affect cognitive abilities will help us gain insight into mechanisms that can constrain cognitive processing and adaptive behavioral responses.

## Introduction

A wide range of species exhibit time- and context-consistent interindividual variation in behaviors, such as activity, exploration, boldness, aggressiveness and sociability, which are all traits related to an individual's personality (1–5). More than 100 years ago, Pavlov was the first to suggest that canine personality could also be a marker for differential performance in associative learning (6). Regardless, potential relationships between animal personality and interindividual differences in cognitive abilities have been relatively understudied in nonhuman animals (7, 8); however, in the last decade, they have attracted new interest across a wide range of taxa, while the causes and strengths of this variation are still under discussion (9).

Proximate factors (attention, selectivity, persistence, and experience) represent behavioral mechanisms that have a major impact on individual learning and cognitive performance (10). Current frameworks that determine behavioral phenotypes that are stable across time and context, such as animal personality (1, 11), temperament (12), coping style theory (13) and behavioral flexibility (14), which are all interrelated (15) have been hypothesized to be functionally related to individual differences in learning and were used to explain consistent within-species interindividual variation in cognitive abilities such as learning and memory. In this way, personality may affect performance in cognitive tasks in that individual variation in attention and encounter rates of environmental stimuli act to either facilitate or inhibit learning (6). It is assumed that natural selection could have parallel effects on cognition and animal personality simultaneously, leading to a correlation between cognition and various personality traits, as both follow a continuum between fast and slow phenotypes (16). The link between variation in personality and cognition forms the basis of the cognitive style hypothesis and is centered on a speed-accuracy trade-off that assumes that individuals may apply different cognitive styles (fast or accurate) based on personality or coping style (7, 17). Proactive individuals, which tend to be bolder, more active, neophilic and fast explorers, learn at higher speeds but at the cost of accuracy. They are likely to become entrenched in a previously learned strategy and thus are less flexible when confronted with new challenges compared to reactive individuals, who are characterized as being rather shy, neophobic, and slow explorers (12, 13, 18). Furthermore, proactive animals are more rigid and fast in decision-making, while reactive animals are thought to be more attentive to environmental changes and stimuli. Attention to environmental cues and cognitive ability are positively related to behavioral flexibility and the ability of an individual to adjust to an ever-changing environment (6, 19). In contrast to much empirical work demonstrating a relationship between personality and learning abilities in different species (20–22), some recent authors cast doubt on a general parallelism between behavioral and cognitive dispositions, as some studies failed to show this link (8, 9, 23–25). For instance, while fast-exploring great tit males (*Parus mayor*) showed more flexible learning abilities than slow-exploring males, female slow explorers outperformed fast explorers, showing a sex dependency in the relationship between personality types and learning (26). Two different zebrafish (*Danio rerio*) strains classified as proactive and reactive were able to learn and recall the fearful association of an associative fear conditioning task. While both coping style strains showed no differences in memory, reactive zebrafish acquired fear memory at a significantly faster rate than proactive zebrafish (27). Another study on narrow-striped mongooses (*Mungotictis decemlineata*) tested the relationship between learning performance, social information and individual differences in boldness: learning performance of seven wild female groups (of two to six individuals each) was tested with an artificial feeding box using a demonstrator-observer paradigm. Bold individuals as well as individuals in groups with demonstrators were faster in learning the task, while individuals without a demonstrator learned the task more slowly, indicating an interaction between personality traits, use of social cues and learning performance (28). In male African striped mice (*Rhabdomys pumilio*), proactive individuals (measured by boldness, activity and exploration) performed better than reactive males in two learning tests: a string-pulling task to obtain food and a door-opening task to reach the nest (29). On the other hand, in guinea pigs and female guppies (*Poecilia reticulata*) exploration, sociability and boldness were not intercorrelated with learning performance (30, 31), again indicating that relationships between personality traits and learning are quite diverse in a number of different species.

In recent years, interest in individual differences in a variety of taxa has increased, including in livestock research (reviewed by 15). There is mounting evidence that these differences play an important role in various contexts in pigs (32–34), cows (35–37), horses (38–40), chickens (41, 42) and goats (43, 44). General knowledge about the cognitive capabilities of livestock (i.e., their ability to acquire, process, store and use information) is of great interest, as cognitive capacities have a major impact on how they are able to interact with their environment (45). Farm animals see themselves confronted with a multitude of challenges (i.e., automated feeding regimes, inflexible housing conditions, rigid management practices) during their lifetimes that have very specific and individual cognitive requirements. To date, numerous studies have focused on quantifying and understanding species-specific cognitive abilities and learning skills in farm animals. For instance, in a visual discrimination task, pigs were able to discriminate between two visual stimuli, and even 1/3 performed well in the reversal task (46). In an acoustic discrimination task, pigs were trained to visit the feeding site after discriminating an individual acoustic signal to obtain a food reward (47). In horses, observers were allowed to watch demonstrators opening a feeding apparatus: While young and lower ranking horses learned the task, older and higher ranking horses did not, showing an age and rank dependency in learning (48). Chickens were able to complete visual occlusion, have biological motion perception and were able to discriminate object and spatial representations, to name a few (reviewed by 42). Meyer and colleagues used an automated learning device that presents artificial symbols *via* a screen and found that goats have clear categorization capacities (49). They were also successful in object permanence tasks (50) and were seeking cognitive challenges by choosing to work for a reward even if they have the possibility of getting it for free, named “contrafreeloading” (51, 52). In playback experiments, it has been shown that goats were able to recall offspring vocalizations for at least 1 year (53, 54). Training a series of visual discrimination tasks showed that goats were able to improve their performance in a learning-to-learn process and develop a learning set (55). In summary, a wide range of cognitive tests has been applied in farm animals to assess a range of cognitive traits. However, differences in cognitive ability between and within individuals of the same species across repeated measurements or across different experimental conditions have also been noted but the causes of these differences remain largely unexplained (7, 8, 45). To date, there is only limited knowledge about a phenotypic link between individual personality and learning capacities in farm animals.

In the current study, we investigated the potential relationship between specific personality traits and learning performance in female dwarf goats. A 4-choice visual discrimination task and three subsequent reversal learning tasks were applied to characterize the learning abilities of the goats. Reversal learning paradigms are commonly used to test behavioral flexibility, a type of phenotypic plasticity that can influence how animals cope with environmental changes. To investigate the relationship between the consistency of certain personality traits and learning, first only data from the first personality test and then the combined data from both personality tests were used separately to test the respective effect on learning performance.

First of all, we expected a relationship between learning and reversal learning in that good learners in the visual discrimination task would need more trials to reach the learning criterion in the reversal learning task and vice versa. This is based on the findings of other studies showing that fast learners exhibited lower behavioral flexibility and, therefore, performed worse in a reversal learning paradigm compared to slow learners (56).

Second, we expected no interrelation between the personality traits deriving only from the first personality test and discrimination learning or reversal learning. On the other hand, taking consistent personality traits into account (data from both personality tests), we expected the more active and bold individuals to disproportionately achieve the learning criterion in the discrimination task faster while inactive and unbold individuals would perform better in the reversal learning tasks, in line with studies regarding behavioral flexibility in the context of coping style (7, 57).

## Animals, materials and methods

### Animals and housing

**Figure 1:**
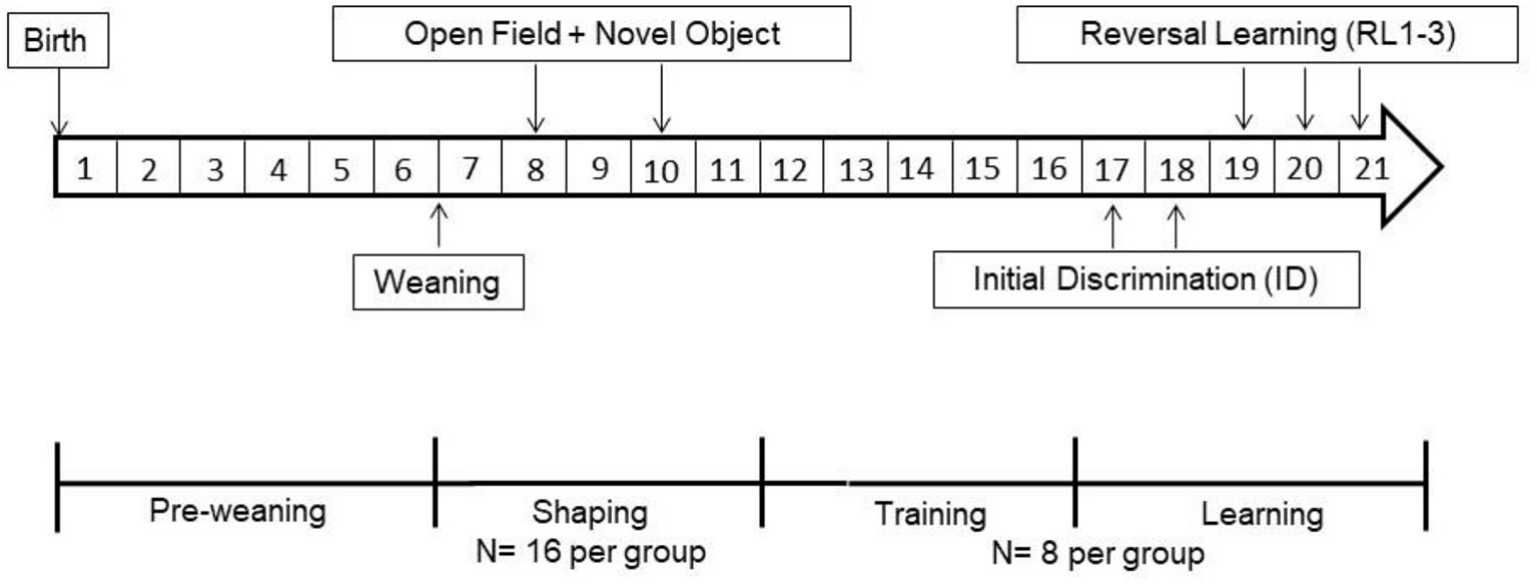
Timeline of the experimental procedure test sequence, housing condition and age of the dwarf goats (given in weeks).

The experiments were conducted with 108 juvenile female Nigerian dwarf goats (*Capra aegagrus hircus*) bred from a line at the Leibniz Institute for Farm Animal Biology (FBN, Dummerstorf, Germany). Due to our breeding program, two experimental runs were conducted per year. From birth to weaning (Figure 1), goats were housed in mixed groups consisting of three to five adult females and their male and female kids. The pens (12 m^2^) were littered with wheat straw as bedding material and contained a hayrack and a round feeder to deliver concentrate (800 g to 1,000 g twice per pen, Vollkraft, Mischfutterwerk GmbH, Güstrow, Germany). Hay and water were provided *ad libitum*. After weaning (seven weeks of age), female goat kids were kept in groups of up to 16 animals per group in pens as described above, with an additional climbing rack as an enrichment activity. All animals were ear tagged and wore a collar with a responder (Urban, Wuesting, Germany) for individual recognition at the free accessible automated learning device. The operating principle of the learning device has been described in detail elsewhere (49). In the following weeks, the kids were shaped to the learning device. During this time, we conducted repeated OF- and NO-tests (Figure 1). At 12 weeks of age, kids were kept in groups of up to eight animals per group in pens as described above. As the number of goat kids was different in every lambing season, runs one and two of the study were conducted with 32 kids each, run three with 14 kids and run four with 30 kids. From here on they will be referred to as goats.

### Behavioral testing

To obtain the respective personality traits, we conducted repeated OF- and NO-tests. Repetitions were performed after 14 days as Takola et al. (58), who conducted a meta-analysis including 115 studies, found that repeatability of responses to novel objects was significant an greater in short-term studies than in long-term studies. In both tests, each goat stayed alone in an arena for 5 min. For both tests, we used an arena (3 × 4.8 m) with opaque walls (2 m high) in a different part of the barn, which was separated from the home pens by two doors. The arena was divided into 12 identical segments (1 × 1.2 m), which were indicated by white lines on the floor. A fully enclosed start box (1 × 1 × 1 m) was connected to the arena and had a guillotine door. For the first NO-test, a traffic cone, and for the second test, a medicine ball was used as the object. To ensure that the object could not be displaced by the focal animal, the object was fixed with a chain hanging from the ceiling. All tests were video recorded (Panasonic WV-CP500). For both tests and both test repetitions, we recorded 82 behavioral measures for each subject coded with Observer XT (Version 12, Noldus Information Technology, The Netherlands). The OF- and NO-tests were conducted on two consecutive days, the same for the repetitions. Further details concerning the testing procedure and the recorded behavioral measures were described by Finkemeier et al. (44).

### Visual discrimination learning

#### Shaping

Over 6 weeks, we shaped the goats stepwise to the learning device. The learning device was integrated into the home pen with free access for all goats 24/7. We used drinking water as a reward (30 ml for each correct choice), which was only accessible at the learning device. For detailed information regarding the shaping procedure, see Langbein et al. (59). In short, we started with a float switch hanging in a water bowl with a button directly above the switch. By pressing the button, the goats could add 30 ml of water to the bowl. After 1 week, the float switch was removed, and we installed first one, later two buttons 20 cm above the bowl, one of which had to be pressed to fill water in the bowl. The reward button was changed weekly and at the end of the shaping phase, daily. At the end of the shaping phase, all goats were able to push the buttons and ensure their water demand independently. According to the veterinary and food control government, the daily water demand of dairy goats ranges between 1.5 and 4 L and based on our experience from previous learning experiments in dwarf goats, we can expect between 23.7 and 28.5 number of drinking actions (30 ml per action), which equals daily water consumption between 0.83 and 0.99 L (51).

#### Training

**Figure 2:**
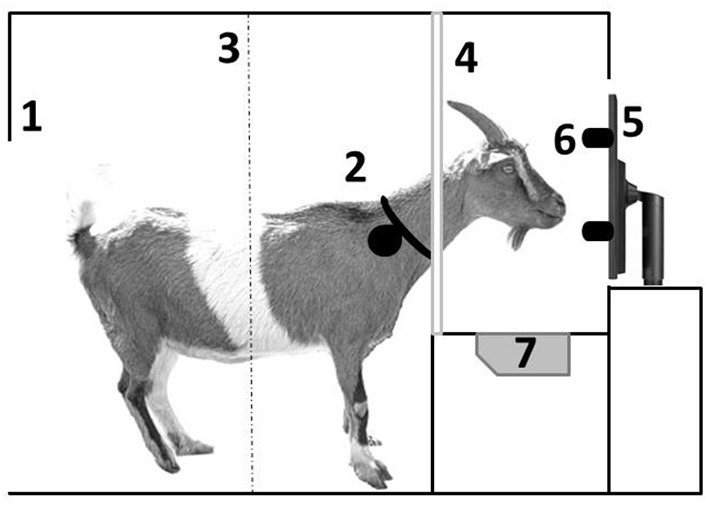
Lateral view of a goat inside the compartment with learning device: 1 = entrance (there is only space for one goat at a time at the device to avoid observational learning by the pen mates); 2 = collar with responder for individual recognition at the device; 3 = light beam indicating when a goat leaves the device; 4 = yoke to put only the head through; 5 = computer screen; 6 = buttons to choose a stimulus; 7 = water bowl for reward dispense.

After completing shaping, goats were regrouped to up to eight animals per group into identically equipped pens, including the learning device. The learning device consisted of a 15-inch LCD screen (resolution of 640 x 480 pixels) behind a transparent acrylic panel. The screen was divided into four virtual sectors. In each sector, we displayed a different symbol (size ~ 7 cm^2^). To allow the goats to choose one of the symbols, four press buttons were mounted on the acrylic panel, one button beside each sector. Figure 2 gives a schematic overview of the compartment with the learning device. For a detailed description of the training procedure, see Langbein et al. (59).

**Figure 3:**
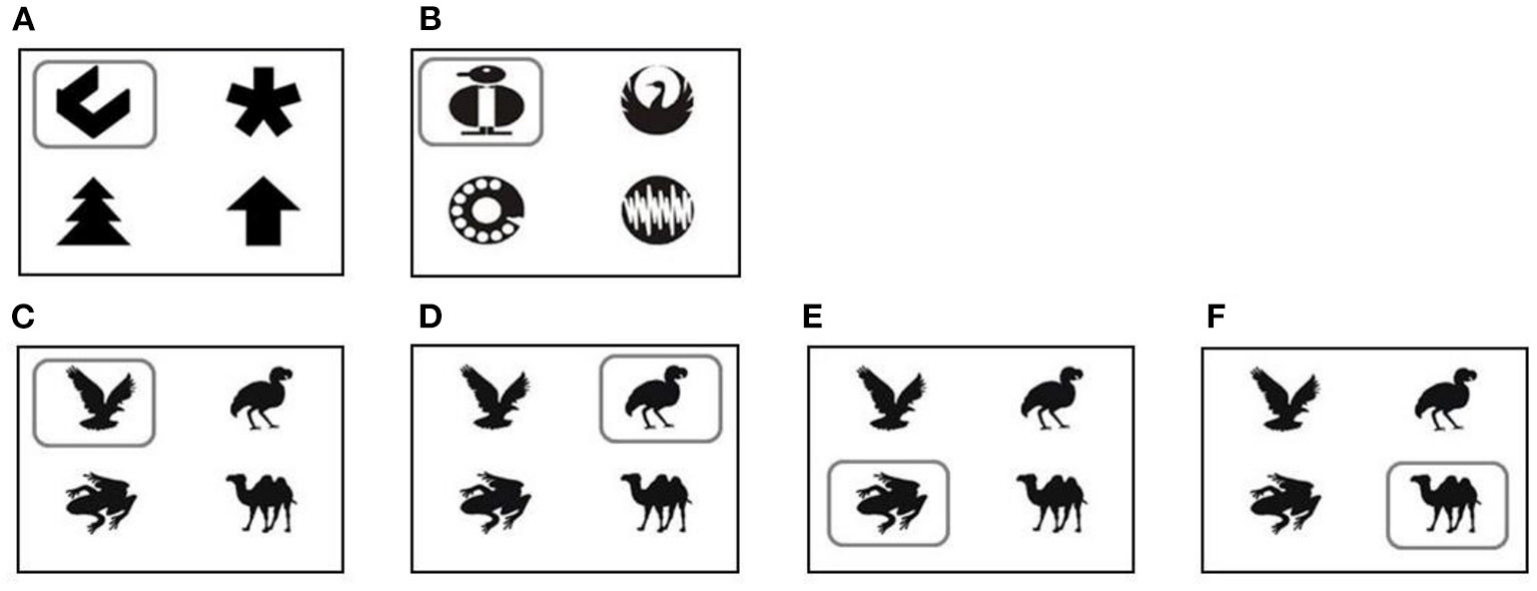
Symbol sets for training: training set 1 **(A)**; training set 2 **(B)**. Symbol sets during learning and reversal learning tasks: Initial discrimination learning task [ID, **(C)**] and the three reversal learning tasks: reversal learning 1 [RL1, **(D)**]; reversal learning 2 [RL2, **(E)**]; reversal learning 3 [RL3, **(F)**]. The rewarded symbols are marked with a square (for illustration purposes only). The position of the different stimuli changed after each individual choice.

During the first week, the goats were presented with a white screen, and all four buttons were rewarded. Next, the goats were presented with the first two training sets (Figures 3A,B). The symbol to learn was marked by a frame. The position of the symbols changed randomly after each trial. Each set was presented for 14 days. Similar to the shaping phase, goats were rewarded with 30 ml water for each correct choice. The device was accessible 24/7 for all goats. Even animals that did not learn the task were able to obtain enough water by increasing the number of trials at the device. At the end of training (60), all goats were able to use the device properly and reached the defined learning criterion (please see the *Data analysis and statistics* section for further details).

#### Initial discrimination learning and reversal learning

To investigate the impact of personality on the learning performance of the goats, a new 4-choice discrimination task was presented (Figure 3C). This initial discrimination task (ID) was trained for 14 days. To investigate the effect of personality on the flexibility of learning behavior, three reversal-discrimination tasks were subsequently presented (RL1-3). The same set of symbols was used as in ID, but in each reversal task, a different one of the three previously unrewarded stimuli was now the stimulus to be discriminated (Figures 3D–F). All other conditions were identical to Training.

### Ethical note

All animal care and experimental procedures were performed in accordance with the German welfare requirements for farm animals and the “Guidelines for the treatment of animals in behavioral research and teaching” (61). All procedures involving animal handling and treatment were approved by the Committee for Animal Use and Care of the Ministry of Agriculture, Environment and Consumer Protection of the federal state of Mecklenburg-Vorpommern, Germany (Ref. 7221.3-2-005/14).

### Data preparation

From 108 tested goats, we excluded those for whom technical issues concerning the learning device occurred (e.g., technical failure of the software and hardware of the learning device and/or loss of transponder for individual identification of the animal.). Finally, a total of 95 goats were included in all further analyses.

The learning performance of the goats was characterized by two steps: First, we analyzed whether the animals reached the learning criterion in ID and in RL1-3, respectively and, if so, we analyzed how many trials they needed to do so (trials to criterion, TTC). Given the four-symbol choice task where chance responding to a given symbol is 25%, the criterion for a statistically significant level (*p* < 0.05) of correct responding was determined according to the Binomial test when *n* = 20 and *p* = 0.25, to be 46% of correct choices in at least two consecutive blocks of 20 trials (learning criterion). This reflects the same learning success compared to the learning criterion of 75% in similar studies applying the common two-choice design (55).

Based on the TTC, we calculated the respective quartiles for the learning performance in ID (Table 1) to test its impact on learning performance in RL1-3. Consequently, Q1 indicated animals with the highest learning performance (lowest number of TTCs) in ID, and Q4 indicated the animals with the lowest learning performance (highest number of TTCs) in ID.

**Table 1:** Calculation of the respective quartiles concerning. (A) the learning performance (number of TTC) in the initial discrimination task (ID); (B) the activity scores based on the results of the principal component analyses of the first open field/novel object test (44); and (C) the boldness scores based on the results of the principal component analyses of the first open field/novel object test. Respective lower and higher limits as well as the category description and number of animals per quartile are indicated.

**Quartile label**	**Lower limit**	**Higher limit**	**Description**	***n*** **(animals)**
**(A) Quartile limits based on the number of TTCs**				
Q1	20	20	“High performer”	24
Q2	40	80	“Good performer”	22
Q3	100	200	“Poor performer”	24
Q4	220	1,700	“Low performer”	24
**(B) Quartile limits based on activity scores**				
A1	−3	−0.5248	“Very inactive”	24
A2	−0.5249	0.0459	“Inactive”	23
A3	0.046	0.6414	“Active”	24
A4	0.6415	3	“Very active”	24
**(C) Quartile limits based on boldness scores**				
B1	−3	−0.9164	“Very unbold”	24
B2	−0.9165	−0.0655	“Unbold”	24
B3	−0.0656	0.7794	“Bold”	23
B4	0.7795	3	“Very bold”	24

The personality traits *boldness* and *activity* were calculated by conducting a principal component analysis (PCA) for the initial OF- and NO-tests and their respective repetitions (please see 44 for further details). We selected 11 behavioral measures (see Supplementary Material) out of 82 identical for both test periods, which were not affected by season and body weight and/or are commonly used in animal personality studies (44, 62, 63). Using these measures, we calculated one PCA for each test repetition (PCA1+PCA2). We found two main PCs in PCA1 (overall MSA = 0.7). The first PC (PC1a; eigenvalue = 3.5) consists of four measures with loadings above 0.7 and below−0.7 describing the interaction with the novel object. The second PC (PC1b; eigenvalue = 2) consists of two measures with loadings above 0.7 and below −0.7 describing active-like behavior. We labeled PC1a “*boldness*” and PC1b “*activity*.” The two PCs explained 32 and 18.2% of the variation in the data, respectively. In PCA2 (overall MSA = 0.71), we found two similar main principal components, with the first describing the interaction with the novel object (PC2a; eigenvalue = 3.7) and the second describing active-like behavior (PC2b; eigenvalue = 1.3), explaining 34% and 12% of the variation in the data, respectively. Finally, we calculated PC scores (from −3 to +3) for each personality trait for each animal in each of the two test periods, where each individual score was calculated from the standardized original data and the respective loadings of each PC separately for PCA1 and PCA2. These scores were used for any further statistical analysis.

We tested the impact of the personality traits on learning performance and characterized *momentary* and *stable* personality traits using either the first or both OF- and NO-tests as the basis for the calculations. Using the personality scores resulting from the PCA1 (*activity* and *boldness* scores from −3 to +3), the respective quartiles were calculated to test the impact of a *momentary* personality trait on cognitive performance (see Table 1 for *activity* and *boldness* quartiles). Conversely, to investigate whether consistency in these personality traits had an effect on learning performance, we selected animals that showed stable *activity* and *boldness* scores in the repeated OF- and NO-tests (= *stable* personality traits). Stability was met if the scores from the first and second OF- and NO-tests were similar (both either positive or negative) for each personality trait (including a protection zone of ± 0.5 from the zero line).

### Statistical analysis

All statistical analyses were conducted in SAS® 9.4 (SAS Institute Incorporated, Cary, North Carolina, USA). The data were evaluated by various analyses of variance (ANOVAs) using the GLIMMIX procedure.

#### Relationship between learning performance and reversal learning

To measure the impact of learning performance in ID, allocated among the four quartiles, on the overall achievement of the learning criterion during RL1-3, we used the variance function for the binomial distribution (binary data) and the logit link function in the statistical model. For more detailed analyses of learning, we ran a model with TTC in RL1-3 as our response variable and evaluated the effect of learning performance in ID (allocated to the respective quartiles, see above), task (RL1, RL2, RL3) and their interaction, which were fitted as fixed effects. Both models contained motherID, pens and season nested in replicates as random effects.

#### Impact of a momentary personality traits on cognitive performance

The impact of the quartiles of *activity* (A1-A4; from very inactive to very active) and *boldness* (B1-B4; from very unbold to very bold) in PCA1 as fixed factors was tested on the number of animals reaching the learning criterion and on the TTC in RL1-3. In each model, motherID, pen and season nested in replicates were included as random effects.

#### Impact of stable personality traits on learning performance

To investigate whether consistency in specific personality traits had an effect on learning performance, we selected animals that showed stable *activity* and *boldness* scores in the repeated OF- and NO-tests. Out of 95 animals, 32 animals showed a stable value in *activity* (16 active, 16 inactive), and 42 animals showed a stable value in *boldness* (16 bold, 26 unbold) across the repeated OF- and NO-tests. The selected animals were then assigned to the groups *stable in activity* (inactive or active) or *stable in boldness* (unbold or bold), which were used as fixed effects on the TTC in ID and RL1-3 in the statistical model. In these models, motherID, replicates and season were included as random effects.

Least squared means (LSMs) and their standard errors (SEs) were computed for the fixed effects in all models, and pairwise differences in the LSM were tested using the Tukey–Kramer correction. All analyses included the animal as a repeated factor in the random statement, and mean differences with *p* < 0.05 were considered significantly different.

## Results

### Relationship between learning performance and reversal learning

**Figure 4:**
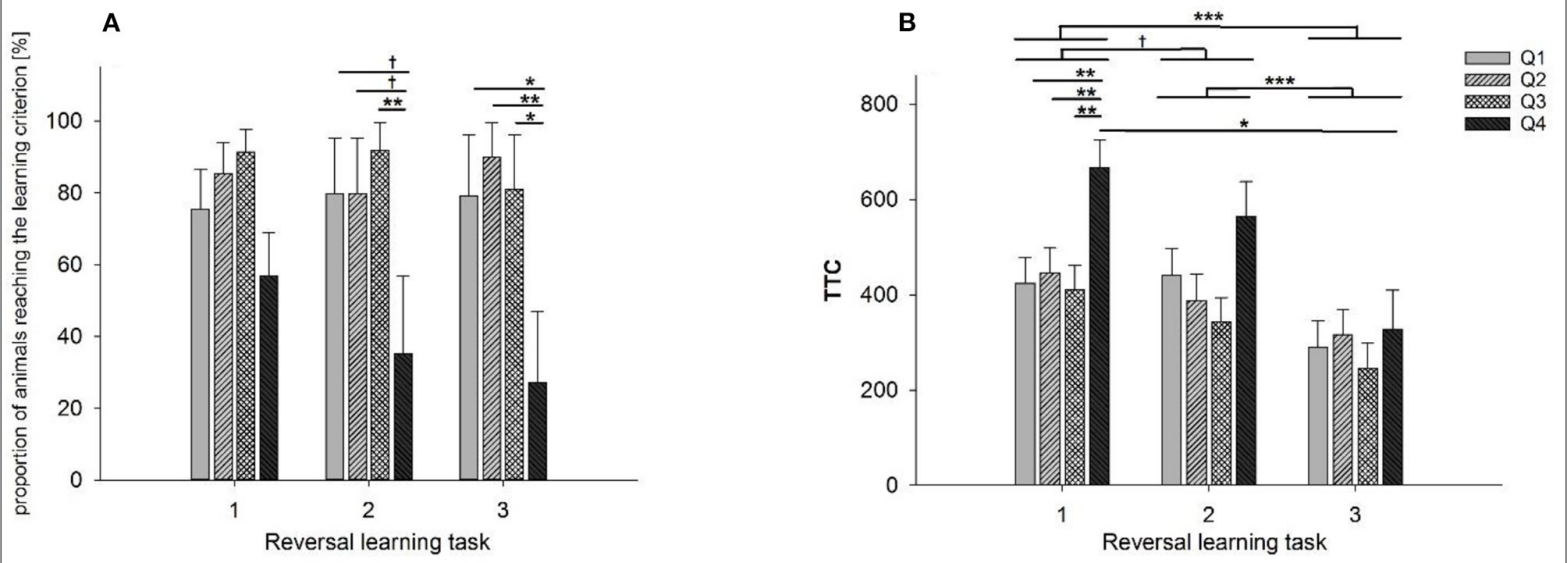
Performance of different groups of goats in the reversal learning task: **(A)** proportion of goats that reached the learning criterion during reversal tasks RL1-RL3. **(B)** Number of trials to reach the learning criterion (TTC) in reversal tasks RL1-RL3. The groups Q1-Q4 indicate the learning performance during the initial discrimination task (ID): Q1: TTC in ID = 20, Q2: TTC in ID = 40–80, Q3: TTC in ID = 100–200, Q4: TTC in ID>220. Data are presented as least squared means and standard errors (LSM ± SE); *p < 0.05, **p < 0.01, ***p < 0.001.

A significant effect of learning performance in ID (Q1–Q4) was found on the number of goats that reached the learning criterion in RL2 (F_3, 41_ = 4.56, *p* < 0.01) and RL3 (F_3, 41_ = 5.05, *p* < 0.01), while a tendency was found concerning RL1 (F_3, 41_ = 2.48, *p* < 0.1). In other words, 46% of the low performers in ID (Q4) were not able to learn the first reversal task (*n* = 11). This effect was significant for the second (71%; *n* = 17) and third reversal tasks (79%; *n* = 19). Goats with the lowest learning performance in ID (Q4) reached the learning criterion in RL2 less frequently compared to goats with only poor learning performance in ID (Q3) (*p* < 0.01) and also tend to reach the learning criterion less frequently compared to goats with good (Q2, *p* < 0.1) and very good (Q1, *p* < 0.1) learning performance in ID. Additionally, low performers in ID (Q4) reached the learning criterion in RL3 less frequently compared to all other groups of learning performance in ID (Q1-Q3), as shown in Figure 4A (Q1 and Q3: *p* < 0.05, Q2: 0.01). Regarding the animals that successfully reached the learning criterion in RL1, RL2 or RL3, we found an effect of learning performance in ID on TTC in the reversal tasks (F_3, 145_ = 5.21, *p* < 0.001). Pairwise comparisons indicate that low performers (Q4) needed significantly more TTC than the other learning groups (Q1-Q3) in RL1 (*p* < 0.01, Figure 4B), while this difference is not apparent in RL2 and RL3 (all *p* > 0.1). Furthermore, we found a significant effect of task on TTC (F_2, 145_ = 20.3, *p* < 0.001) in that the animals significantly reduced their TTC in RL3 compared to RL1 and RL2 (*p* < 0.001, respectively) that was mainly apparent in Q4 regarding the TTC in RL3 compared to RL1 (*p* < 0.05). However, the interaction between learning performance in the ID and RL tasks revealed no significant effect.

### Impact of momentary personality traits on learning performance

The statistical analysis indicated no significant effects of *activity* (A1-A4) or *boldness* (B1-B4), based on only the first conducted OF- and NO-tests, on the number of goats that reached the learning criterion during RL1-3. Furthermore, *activity* and *boldness* did not show any significant effects on the TTC either during ID nor during RL1-3.

### Impact of stable personality traits on learning performance

**Figure 5:**
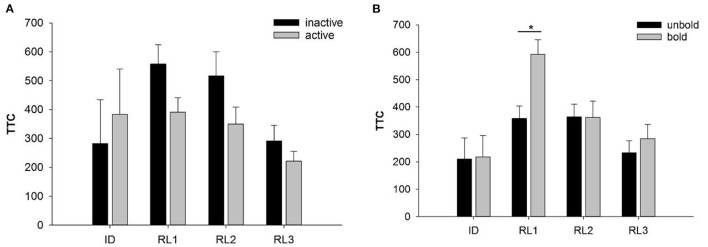
Performance of goats with stable personality scores in activity **(A)** or boldness **(B)** in the reversal learning tasks: **(A)** number of trials to reach the learning criterion in the reversal tasks RL1-RL3 of goats assigned to the inactive (black bars) vs. active groups (gray bars). **(B)** Number of trials to reach the learning criterion in the reversal tasks RL1-RL3 of goats assigned to the groups unbold (black bars) vs. bold (gray bars). Data are presented as least squared means and standard errors (LSM ± SE); *p < 0.05.

We found no significant effects of *stability* in *activity* or *boldness* on the number of goats that reached the learning criterion in RL1-3. Regarding TTC, *stability in activity* (inactive vs. active) was not found to have a significant effect on TTC in ID or RL1-3 (Figure 5A). In contrast, *stability in boldness* (unbold vs. bold) significantly affected TTC in RL1 (F_1, 7_ = 11.36, *p* < 0.05). As shown in Figure 5B, bold animals exhibited a lower learning performance (higher number of TTCs) in RL1 than unbold animals. This effect was not observed in RL2 and RL3.

## Discussion

Our study investigated the potential relationship between specific personality traits and individual learning performance by using a visual discrimination paradigm. Goats learned to associate a visual stimulus with a reward in the initial 4-choice visual discrimination task (ID) presented 24/7 using an automated learning device and were able to reverse their associations in a serial reversal task (RL1-3). Their performance in the three consecutive RL's depended on their learning success in the ID in that low performers in the ID also performed low in the RL's. Taking specific personality traits (measured once) into account, we did not find any evidence of a relationship between *activity* and/or *boldness* and learning performance, either in the discrimination task or in the reversal tasks. However, including only the animals that were *stable* for personality trait *activity* and/or *boldness* (measured twice), no evidence for any link between *activity* and learning performance was found, while *boldness* revealed a significant link to learning performance in the first reversal task in that unbold individuals outperformed bold individuals in RL1.

The learning performance of the goats in the three consecutive reversal tasks depended on their learning success in the initial learning task. A higher proportion of goats performing low in the initial discrimination task did not achieve the learning criteria in the reversal tasks compared to the goats that learned the initial discrimination task better. This effect is not as obvious in the first reversal task but reaches significance in the second and third reversal tasks. Additionally, the low-performing goats that were able to reverse the initially learned association needed more trials to solve the reversal task, which was significant at least in RL1. This indicates a relationship between visual discrimination learning and reversal learning in goats in the sense that the learning speed in the visual discrimination task would predict the learning performance in a reversal task regarding low-performing learners. Unexpectedly, goats who performed low in the initial discrimination task also performed low in the reversal task. This finding contrasts other learning studies showing that good learners (high learning speed) seem to be poor reversal learners (low learning speed), which has been demonstrated in guppies (64) and parrots (65). Discrimination learning underlies the ability to associate a stimulus with appetitive (or aversive) stimuli, whereas reversal learning involves the capability of extinguishing a previously learned association to form a new association. This is thought to be more cognitively challenging, as previously learned cues must be deleted before a new association can be formed. Reversal learning tasks are frequently used as a measure of behavioral flexibility, as they require the subjects to flexibly adjust their behavior when the reward-related contingencies that they have previously learned are reversed (66). The clear difference between low performers and the other three learning groups in learning performance may indicate less flexibility and greater perseverance in low performers once they understood the initial discrimination task. One possible explanation comprises the role of the individual stress level during learning. A range of studies have demonstrated that elevated corticosterone levels affect learning performance across taxa, although the degree to which learning is affected and whether exposure results in benefits or decrements depends on the extent and timing of corticosterone exposure (67–69). The low-performing individuals in our study may be more stressed compared to the other three groups of learners due to the stress originating from the learning task itself or from external factors such as dominance or rank within the group. These factors have already been found to show a relationship to learning performance and might have impaired learning performance in the initial discrimination task. To shed light on this issue, and as we did not investigate the individual stress level in our study, future research should take into account the stress responses of animals in such learning tests. However, the learning performance of the other three learning groups in the discrimination task did not have an effect on reversal learning; thus, we did not find a negative correlation, as has been shown in red junglefowl (70). The learning groups might differ in several aspects associated with performance, such as motivation or physical strength. We also take into account differences in general learning ability, as some animals might be more cognitively impaired than others, which is likely to explain the differences observed in discrimination and reversal learning. Furthermore, goats seem to be able to improve their performance across three reversals by progressively reducing their TTC, indicating that they are getting better at switching to the alternative stimulus. Similar serial reversal learning experiments, in which the alternation of training to criterion and contingency reversal was repeated several times, have revealed that rats, pigeons, frogs and goats improve their performance over successive reversals in a learning-to-learn process (71–73). In our study, we found that low performers were able to improve their TTC across the reversal tasks, but we must note that goats that did not achieve the learning criterion at all in RL1-3 fell out of scope.

Taking specific personality traits into account, we did not find any evidence of a relationship between the two *momentary* personality traits *activity* and *boldness* (measured once) and learning performance either in the discrimination task or in the reversal tasks. We might have expected, based on previous studies concerning personality measures and learning/cognition, that bold and/or active individuals learn to associate a cue with a reward faster than unbold and/or inactive individuals (7, 74) but perform lower in reversal learning (56, 75, 76). A possible explanation for the lack of a link between personality traits and learning success in this analysis may be that only a single measurement of personality was integrated in the analysis. A recent review stated that personality measured just once cannot provide enough information about all aspects of personality differences (77). This is easily comprehensible, as animal personality refers per definition to the repeatable part of an individual's behavior (12, 78), and a behavior measured only once probably reflects mostly the within-individual rather than the among-individual component (77, 79). Among studies that have investigated the relationship between personality and cognition in nonhuman animals, some conducted a behavioral test only once to characterize specific personality traits and often failed to show interrelations between personality and learning, while it is recommended to measure personality traits across time and contexts by applying several repetitions of different tests to find a connection between personality and learning capabilities (28, 31, 80). For example, Christensen et al. (81) found that behavior toward a novel object (labeled exploration) correlated with learning performance in a visual discrimination task in horses. More exploratory horses were more successful in a two-choice visual discrimination task (81). Similar to our study, the NO-test was conducted repeatedly, indicating that relationships between cognitive performance and personality can be found when personality traits are confirmed to be stable over time because they are measured at least twice.

In our study, the behavior of the individuals was observed in two tests that were independent from each other, and two separate PCA were conducted. In a following step, we used the data from both personality tests, including only the animals that were stable for personality trait *activity* and/or *boldness*, to test whether stability in a specific personality trait would have an effect on learning performance. We found no evidence for any link between *activity* and learning, either in ID or in RL, whereas *boldness* revealed a significant link to learning performance in RL1. By exhibiting a higher level of activity and exploration, active personality types sample their environment more rapidly, albeit more superficially. As a consequence, these animals should learn novel tasks more quickly but at the cost of accuracy and responsiveness to changes in the meaning of cues (6, 13, 57). Caused by their superficial sampling and inaccuracy, they were thought not to perceive the change in cue meaning during reversal learning and thus would make a greater number of mistakes when associating the symbols with the reward. In contrast, inactive types were thought to have a reduced learning speed but reveal more learning flexibility through their greater attention toward cue relevancy, increasing their accuracy under variable environmental conditions. We therefore expected active individuals to successfully reach the learning criterion in the discrimination task more rapidly and that inactive individuals would outperform active individuals in the context of reversal learning tasks. In testing these predictions, we found that *activity* did not have any influence on learning success in the goats. Similar results were reported by Chung et al. (17), where active (“fast”) lizards did perceive a change in cue meaning (other than expected) and were not less accurate than their inactive (“slow”) conspecifics when tested in a two-phase associative task. In general, several terms in the context of animal personality research have referred to partially overlapping concepts (82), and traits such as exploration, boldness (and sociability) are most commonly used in the context of animal personality. Only a few studies find a direct relationship between activity and learning success, and if so, they reveal different results (17, 74, 83). This may be due to varying definitions and measurements of activity. While some studies measure activity as the number and rate of transitions between squares (17; lizard) or swim distance (83; guppies), others use locomotion behavior that results in a change in body position in space as an indicator for activity (74; cavies). This impairs the general comparability across experimental studies, measurement methods and statistical analyses. There is some evidence that links between activity and learning abilities are highly task dependent. For example, fearful and active individuals perform better in an avoidance task, whereas the authors did not find such a relationship in a backwardss-forwards task in horses (84). It is therefore possible that activity might reveal a predisposition to react to specific stimuli involved in learning.

However, we found a significant impact of the *stable* (repeatedly measured) personality trait *boldness* on learning performance in RL1 (unbold individuals outperform bold individuals) but not in ID. This partly reflects the general assumption that bold or proactive individuals are better at learning a discrimination task, but unbold or reactive individuals are better at adapting to changes in an already learned task reflecting a higher behavioral flexibility (7, 57, 76, 85–87). This association has been shown in studies with great tits (26) and red junglefowls (88), while others fail to show this relationship (31). Differences in the readiness to approach a novel stimulus have been described as risk-taking behavior, novelty-seeking, proactivity or boldness in different studies across different species and have been shown to be a distinguishing factor between individuals differing in coping patterns or personality traits (13, 89–91). It is assumed that bold/proactive animals are more successful in forming routines during learning, which results in being less able to react flexibly in adapting their behavior to changing environmental conditions (20, 92, 93). Interestingly, the difference in learning success only refers to the first reversal task and disappeared in RL2 and RL3, as bold individuals were able to improve their learning performance. We assume that they learned to form a new routine of the concept of reversal, strengthening the hypothesis that bold individuals are fast learners in the sense that they quickly form routines. This interpretation fits with Cockrem's (92) classification of personalities in that proactive individuals may perform better in environments that are constant or predictable compared to reactive individuals who perform better in unpredictable conditions. Reactive individuals may tend to generalize a formerly learned rule (i.e., “one of the symbols is rewarded”) more quickly being able to shift the general rule to a new symbol. On the other hand, fast explorers may learn more about the absolute properties of the stimulus in the visual discrimination task and thus fail to classify new symbols immediately “correctly.” Once the reward reversal was no longer new (RL2 and RL3), proactive individuals learned that the general concept changes and were able to form a new routine. Even so, it is unclear why and how learning more or less flexibly is related to how individuals react to a novel object in the context of a standardized behavioral test. At least, novelty seems to play an important role in driving these processes, as differences between individuals refer to both the novel object and the novel (first) reversal learning task. In most studies, the trait boldness refers to behavioral reactions to novel stimuli and situations and is often measured in a NO- or novel human test (reviewed by 8). However, boldness has also been used in predator-dependent contexts showing a relationship to learning performance, whereas boldness measured in a NO-test did not, especially when measured only once (reviewed by 9). Furthermore, boldness concepts may refer to an individual's reactions to risky situations (12, 94), while exploration is often used in the context of an individual's reactions to new situations (12). The differences between a new and/or risky situation are not easily detectable and depend to a large amount on the type of experimental setup and recorded parameters (12, 18, 82).

To address our findings, the performance of the goats in the three reversal tasks depended on their learning success in the initial discrimination task in that low performers in the initial discrimination task also performed low in the reversal tasks. We found evidence in the current study that there is a relationship between learning and *boldness* in goats: bold individuals show reduced behavioral flexibility, as shown in the first reversal task; however, bold animals were able to adapt in the subsequent reversals once a new routine was formed.

## Conclusion

To conclude, in the present study, one personality trait, *boldness*, proved to have substantial consequences on learning performance in a reversal task. Our findings show that we should take personality bias into account when conducting learning or cognitive experiments and underline the importance of the repeated measurement of individual personality traits. However, the explanation for the observed interrelations between personality traits and learning performance is currently unclear and requires further empirical and theoretical investigation. To understand whether intrinsic differences in learning exist across individuals, we need to investigate the extent to which learning and personality traits covary and identify the mechanisms that can constrain cognitive processing and adaptive behavioral responses.

## Data availability statement

The datasets presented in this study can be found in online repositories. The names of the repository/repositories and accession number(s) can be found below: https://osf.io/wudj7/?view_only=ed372f7bdded4ff2a46901a0bdc15081.

## Ethics statement

The animal study was reviewed and approved by Committee for Animal Use and Care of the Ministry of Agriculture, Environment and Consumer Protection of the federal state of Mecklenburg-Vorpommern, Germany (Ref. 7221.3-2-005/14).

## Author contributions

JL conceptualized the study. M-AF and AK collected and analyzed the data and did the statistical analysis together with AT. M-AF, AK, JL, and BP contributed to the conception of the manuscript. M-AF, AK, and JL wrote the manuscript. All authors contributed to the article and approved the submitted version.

## Funding

This work was supported by the Deutsche Forschungsgemeinschaft (DFG, ME 4280/1-1).

## Conflict of interest

The authors declare that the research was conducted in the absence of any commercial or financial relationships that could be construed as a potential conflict of interest.

## Publisher's note

All claims expressed in this article are solely those of the authors and do not necessarily represent those of their affiliated organizations, or those of the publisher, the editors and the reviewers. Any product that may be evaluated in this article, or claim that may be made by its manufacturer, is not guaranteed or endorsed by the publisher.
