# Supplementary material for: Personality traits affect learning performance in dwarf goats (Capra hircus)
Source: Front Vet Sci. 2022 Jul 15;9:916459. doi: 10.3389/fvets.2022.916459 (PMC9336648; doi:10.3389/fvets.2022.916459)
Supplement: Supplementary file 1 [file Table_1.docx]

***Supplementary material***

**Table 1:** Definition and type of recording of 11 measures of 7 behaviours during the open-field (OF) and novel-object (NO) tests used for both PCAs; D = duration (total time in s), F = frequency, L = latency (time in s until behaviour was first shown). This table is redrawn from Finkemeier et al. (44).

| **Behaviour** | **Type of recording** | **Definition** |
| --- | --- | --- |
| General activity  (OF +NO) | D | Max. 3 legs touching the ground, forward movement |
| Rearing up  (OF + NO) | D | Animal stands on both hindlegs on the ground and touches the walls of the arena with the forelegs |
| Time spent in corner segments | D | Time that the animal spends in the corner segments of the arena |
| Time spent in inner segments | D | Time that the animal spends in the inner segments of the arena |
| Vocalizations | F | Any kind of bleats the goat makes |
| Time spent in the segments near the object | D | With at least the forelegs in the segments (5 in total) which are near the object |
| Contacts with object | D, F, L | Snout contact with the novel object |
